# Characterization and Description of the Fecal Microbiomes of Pet Domestic Ferrets (*Mustela putorius furo*) Living in Homes

**DOI:** 10.3390/ani13213354

**Published:** 2023-10-29

**Authors:** Elisa Scarsella, J. Skyla Fay, Guillaume Jospin, Jessica K. Jarett, Zhandra Entrolezo, Holly H. Ganz

**Affiliations:** 1 AnimalBiome, 400 29th Street, Suite 502, Oakland, CA 94609, USA; elisa@animalbiome.com (E.S.); guillaume@animalbiome.com (G.J.); jess@animalbiome.com (J.K.J.); zhandra@animalbiome.com (Z.E.); 2 Ferret Microbiome Research Institute, Hull, MA 02045, USA; skyla.fay@gmail.com

**Keywords:** domestic ferret, *Mustela putorius furo*, gut, microbiome, carnivore, domestic cat, *Felis catus*

## Abstract

**Simple Summary:**

This study compares the gut microbiomes of healthy domestic ferrets and cats, which are both carnivorous pets. While ferrets have a population of around 500,000 in the United States, little is known about their gut bacteria. We collected stool samples from 36 healthy ferrets and 36 healthy cats and analyzed their bacterial DNA. We found that the ferrets had more Firmicutes and Proteobacteria, whereas the cats had higher levels of Bacteroidota and Actinomycetota. The ferret microbiomes had lower diversities. Specific bacterial genera like *Clostridium*, *Streptococcus*, *Romboutsia*, *Paeniclostridium*, *Lactobacillus*, *Enterococcus*, and *Lactococcus* were associated with the ferrets. The significant differences in the microbiomes between ferrets and cats suggest unique gastrointestinal care requirements for ferrets, particularly if they have digestive issues. Future studies should explore how the gut microbiomes of sick ferrets differ from healthy ones and how they respond to diet and medical treatments.

**Abstract:**

The domestic ferret (*Mustela putorius furo*) is a popular companion pet in the United States, with an estimated population of 500,000. Despite being obligate carnivores with a fast digestive system, little is known about their gut microbiomes. This study aims to compare the fecal microbiomes of healthy domestic ferrets and cats, which are both obligate carnivores. We collected and analyzed stool samples from 36 healthy ferrets and 36 healthy cats, sequencing the V4 region of the 16S rRNA gene. Using QIIME 2, we assessed the alpha and beta diversities and identified the taxa differences. Compared to cats, ferrets exhibited a higher representation of Firmicutes and Proteobacteria, while Bacteroidota and Actinomycetota were more prevalent in cats. The ferrets’ microbiomes displayed lower alpha diversities. The highly present bacterial genera in the gut microbiomes of ferrets included *Clostridium sensu stricto*, *Streptococcus*, *Romboutsia*, *Paeniclostridium*, *Lactobacillus*, *Enterococcus*, and *Lactococcus*. Notably, the ferrets’ microbiomes significantly differed from those of cats. This research highlights the potential differences in gastrointestinal care for ferrets, emphasizing the need for tailored approaches. Future studies should explore microbiome variations in ferrets with gastrointestinal issues and their responses to dietary and medical interventions.

## 1. Introduction

The domestic ferret (*Mustela putorius furo*) is an obligate carnivore belonging to the *Mustelidae* family that is closely related to minks, weasels, and otters [1]. Due at least in part to their affectionate nature, small size, handsome markings, and ease of care, ferrets have become increasingly popular as companion animals. In 2018, 326,000 households in the United States were thought to have a pet ferret, with a total estimated population size of ~ 500,000 individuals in the United States, where it is considered a specialty or “exotic” pet [2] (AVMA 2018), and this number is likely to have increased since the pandemic.

Although there are many studies related to the gut microbiomes of other companion animals, particularly cats and dogs (e.g., [3,4,5,6]), much less is known about the ferret gut microbiome, particularly of those living in homes. While a few studies have investigated the microbiomes of ferrets, all were performed on ferrets living in colonies [7,8,9,10]. None have characterized the microbiomes of pet ferrets living in homes. The living environment has a large effect on the fecal microbiome composition. For example, pet cats living in homes differ significantly from those in animal shelters and have a greater portion of their microbiomes represented by core bacterial taxa [11].

In a recent review of nutritional research for pocket pets, Bullen (2021) posited that as an obligate carnivore, the fecal microbiomes of domestic ferrets would be expected to have lower complexities and alpha diversities compared to herbivores, such as rabbits and rodents [12], where microbial fermentation plays an essential role in the processing of plant material within the digestive tract. In keeping with their carnivorous diet, ferrets have very short gastrointestinal tracts. The small intestine is the longest part of the digestive system, and it is nearly 20× longer than the large intestine [13]. Due to their very simple gastrointestinal (GI) tract, the GI transit time in the domestic ferret is very fast, ranging from 148 to 219 min when fed a meat-based diet [14] and when fed a dry cat food diet [15], respectively. Beyond this, the roles of diet type and composition in the food transit time has not been explored to date [16].

A comparative study of laboratory animals in China contrasted the compositions of the gut microbiomes of domestic ferrets with those of marmosets, woodchucks, mini pigs, tree shrews, and humans and found that the ferret microbiome has a larger representation of bacteria in the phylum Proteobacterium than the other species [10]. A less recent study attempted to characterize the intestinal microbiomes of ferrets through bacterial culture techniques [7]. The presence of anaerobic and microaerophilic bacteria was evaluated in fresh fecal specimens of 40 ferrets in apparently good health. The most frequently isolated anaerobic bacterial strains were *Clostridium acetobutylicum*, *Bifidobacterium bifidum*, *Lactobacillus acidophilus*, and *Actinomyces neaslundii*. They also found *Bacteroides ureolyticus*, now placed in the genus *Campylobacter*, and *Clostridium perfringens*, although the ferrets were all in good health. The microaerophilic bacteria that were isolated included *Helicobacter* spp., *Campylobacter* spp., and *Shewanella putrefasciens*. The latter was found only in ferrets bred in Naples, Italy, and given its environmental origin, it is assumed that it derives from drinking water in this area.

The domestic ferret is also used in biomedical research as a model for several human gastrointestinal diseases [14]. One example is related to infections with *Helicobacter mustelae*, one of the more common bacterial diseases in domestic ferrets that causes ulcerative gastritis and duodenitis. Ferrets are used as research models for human gastritis caused by *Helicobacter pylori*, because *H. mustelae* is antigenically related to *H. pylori*. Moreover, physiologic stress with excess cortisol levels can cause *Helicobacter* overgrowth, leading to ulcers in ferret study population models. Ferrets are also used as animal models for several GI diseases, like *Helicobacter* gastritis, gastric carcinoma, pyloric and intestinal ulceration, Inflammatory Bowel Disease (IBD), colitis, and gastrointestinal neoplasia. Together, these conditions result in various forms and degrees of diarrhea. IBD can have multiple causes and may have a genetic component. It can be challenging to diagnose due to clinical signs that are similar to other enteric diseases. Insulinoma, a functional insulin-secreting beta-cell tumor, is one of the three most common tumors in ferrets [17]. In most cases, the gold standard to diagnose these pathologies is intestinal biopsies, together with clinical signs, although the cost is expensive, and the surgery is invasive. Ferrets have also been used as model animals for studying psychiatric illnesses, particularly autism spectrum disorder [8,9].

Many studies have demonstrated that the gut microbiomes of humans and animals are affected by the health status of the host and other environmental factors [3]. Moreover, the microbial population that composes the gastrointestinal tract (GI) has been shown to be fundamental for metabolic activities, the protection against pathogens, and signaling to the immune system [4]. Diet modulation has been demonstrated to have a strong impact on the gut microbiomes of healthy subjects [5,18,19,20], although it has been underpinned by high individual variability [21]. Nevertheless, there is a group of highly prevalent bacteria in the fecal materials of healthy hosts, suggesting the existence of a core gut microbiome [22].

Several studies, particularly studies on humans, have highlighted the need to focus on the gut microbiome in order to understand the mechanisms of interactions between the microorganisms and the functions that those bacteria have on gastrointestinal disease [23,24]. Moreover, a focus on the microbial population of the GI tract opens a new way to treat animals, especially dogs and cats, for IBD and chronic gastro-enteropathies [25,26]. The field of the gut microbiome in companion animals is growing, but to the best of our knowledge, this is the first characterization of the gut microbiome in a population of healthy pet ferrets. The aim of this study is to fully describe the bacterial compositions of the fecal microbiomes of healthy pet ferrets living in homes, and to compare them with the gut microbiomes of healthy pet cats. Because of their relative rarity as companion animals, ferrets are sometimes treated with diets and medications that are designed for domestic cats or dogs. However, due to large differences in the anatomy and physiology of their digestive tracts, we anticipate that the microbiomes of domestic ferrets will differ substantially from those of domestic cats. These differences may be meaningful when navigating gastrointestinal and other health conditions potentially modulated by the gut microbiome.

## 2. Materials and Methods

### 2.1. Animals and Sample Collection

Samples from thirty-six apparently healthy domestic ferrets and thirty-six apparently healthy domestic cats were collected from pet owners. Animals were reported as healthy by the pet owners when they were without any symptoms and because they were not under any therapeutic treatment at the time of sample collection. Fecal samples from healthy ferrets were recruited from eight different households across the US. Both females (14 ferrets, 5 of which were spayed) and males (21 ferrets, 11 of which were neutered) were included in this study, and the cats were mostly mixed breeds. All of the animals were adults. The cats were a subgroup, selected randomly, from a collaborative research project called “Kittybiome: kitty microbiomes for cat health and biology”, which aimed to characterize the gut microbiomes of domestic cats. The full dataset is described in Ganz et al. [11]. Both ferrets and cats recruited for this study were healthy based on no evident signs of a health condition reported by the owners, in order to avoid bias in the comparison of the gut microbiomes. Ferrets with prior exposure to antibiotics were excluded from the study. Information about name, date of birth, body weight, spay or neutered status, and diet were collected with a survey (Supplementary Table S1). Right after defecation, ferret and cat stools were collected in sample vials (2 mL screw-cap tubes) containing 100% molecular-grade ethanol and extracted upon receipt.

### 2.2. Sample Processing

Genomic DNA was extracted from all samples using the 100-prep DNeasy PowerSoil Pro DNA Isolation kit (Qiagen, Hilden, Germany). Samples were placed in a PowerBead Pro Tube containing CD1 solution and bead beaten for five minutes, followed by centrifugation at 15,000× *g* for one minute. The remaining extraction protocol was performed as per the manufacturer’s protocol. DNA concentration was recorded using a QUBIT dsDNA HS Assay (Thermo Fisher, Waltham, MA, USA). Amplicon libraries of the V4 region of the 16S rRNA gene (505F/816R) were generated using a dual-indexing one-step PCR with complete fusion primers (Ultramers, Integrated DNA Technologies, Coralville, IA, USA) with multiple barcodes (indices) [27]. PCR reactions containing 0.3–30 ng template DNA, 0.1 μL Phusion High-Fidelity DNA Polymerase (Thermo Fisher, Waltham, MA, USA), 1× HF PCR Buffer, 0.2 mM each dNTP, and 10 μM of the forward and reverse fusion primers were denatured at 98 °C for 30 s; cycled 30 times at 98 °C for 10 s, 55 °C for 30 s, and 72 °C for 30 s; incubated at 72 °C for 4 min 30 s for a final extension; and then held at 6 °C. PCR products were assessed by running on 2% E-Gels with SYBR Safe (Thermo Fisher, Waltham, MA, USA) with the E-Gel Low Range Ladder (Thermo Fisher, Waltham, MA, USA), and then purified and normalized using the SequalPrep Normalization Kit (Thermo Fisher, Waltham, MA, USA) and pooled.

The final libraries were quantified with QUBIT dsDNA HS assay (Thermo Fisher, Waltham, MA, USA), diluted to 1.5 pM, and denatured according to Illumina’s specifications for the MiniSeq. Identically treated phiX was included in the sequencing reaction at 25%. Paired-end sequencing (150 bp) was performed on the MiniSeq (Illumina, San Diego, CA, USA).

### 2.3. Sequence Data Processing

The Quantitative Insights into Microbial Ecology (QIIME 2, version 2021.8) [28] was used to process the raw sequences. The sequences related to the ferret samples were uploaded to the NCBI Sequence Read Archive (Bioproject ID PRJNA988157). After demultiplexing, sequenced reads that passed the quality check (Phred score ≥ 30) were annotated for 16S rRNA against version 138 of the SILVA reference database, with 99% identifying with reference sequences. Chimeras were also detected and removed by filtering, and the remaining sequences were clustered into exact sequence variants by using an open reference approach in QIIME 2. This procedure is the preferred strategy for exact sequence variant selection in QIIME 2, which includes taxonomy assignment, sequence alignment, and tree-building steps.

### 2.4. Computational and Statistical Analyses

Data were analyzed using QIIME 2 [28]. The number of reads was rarefied to 5000. Briefly, alpha diversity was calculated using Shannon index, according to the equation H’ = −sum (Pi × ln Pi), where Pi = frequency of every genus within the sample, and Evenness index was calculated as J’ = H’/lnS, where S is the total number of genera within each sample. The comparison between groups was performed with a non-parametric Kruskal–Wallis test. Beta diversity was evaluated using the phylogeny based on both weighted and unweighted UniFrac distances [29]. Beta diversity was visualized using Principal Coordinate Analysis (PCoA) plots.

The 16S rRNA annotated sequences were normalized to % abundances profiles. A linear discriminant analysis (LDA) of the effect size (LEfSe), with an LDA score threshold of 2.0, was applied to detect taxa that differed between healthy ferrets and healthy cats [30].

Gneiss analysis [31] was used to identify differentially abundant taxa among groups. To facilitate the analysis, the taxa with a per-sample frequency lower than ten reads were filtered out. Principal balances were obtained via Ward’s hierarchical clustering using the correlation-clustering command. Log ratios between groups at each node of the tree were calculated using the ilr-hierarchical command. A multivariate response regression model was created by running a linear regression separately on each balance using the ols-regression command. The contributions of ferrets vs. cats as covariates to the overall community variation were visualized through a regression summary and dendrogram heatmaps. Balances significantly affected by the covariates were identified as those with a *p*-value of less than 0.05.

## 3. Results

**Figure 1:**
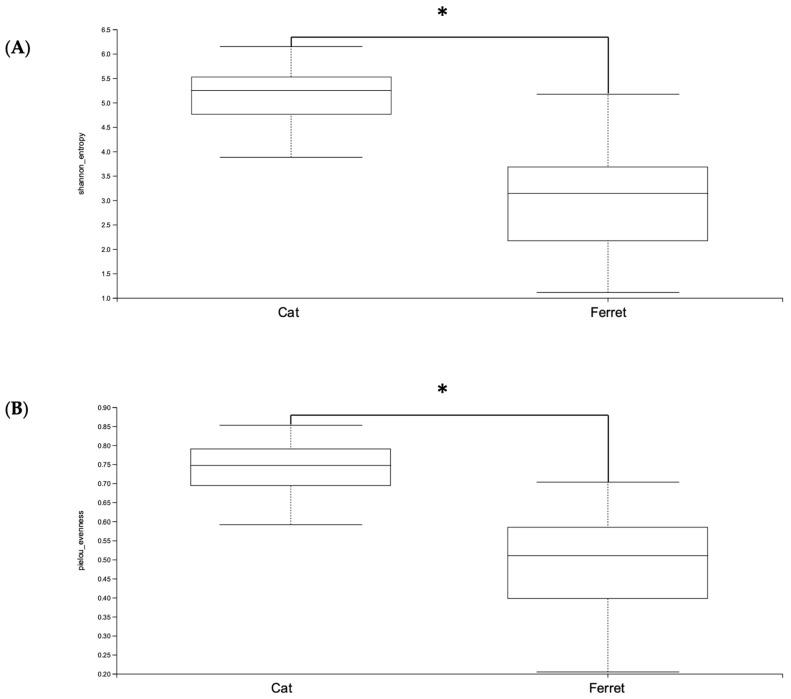
Alpha diversity—(**A**) Shannon and (**B**) Pielou’s Evenness indexes—of Amplicon Sequence Variants (ASVs) of healthy ferrets and healthy cats. A Kruskal–Wallis non-parametric test was applied, and significant differences were determined from *p* < 0.05. * denotes *p* < 0.05.

The ferrets’ gut microbiomes were composed mainly of Firmicutes, Proteobacteria, and Actinobacteriota (Table 1). Bacteria belonging to the phylum Firmicutes were the majority, with a Relative Abundance (RA) of 90.4%, followed by phylum Proteobacteria (4.8%) and phylum Actinobacteriota (3.2%). This was notably different from the composition of the gut microbiomes of cats, since the main phylum was still Firmicutes, but with an RA of 64.6%, followed by Bacteroidota (19.2%) and then Actinobacteriota (10.1%). The taxonomic designations of the core fecal microbiomes of healthy pet ferrets are shown in Table 2. These include all bacteria found in at least 50% of the ferret samples. The microbiomes of the healthy cats had significantly greater alpha diversities in terms of both the Shannon and Evenness indexes compared to the healthy domestic ferrets (*p* < 0.05) (Figure 1).

**Figure 2:**
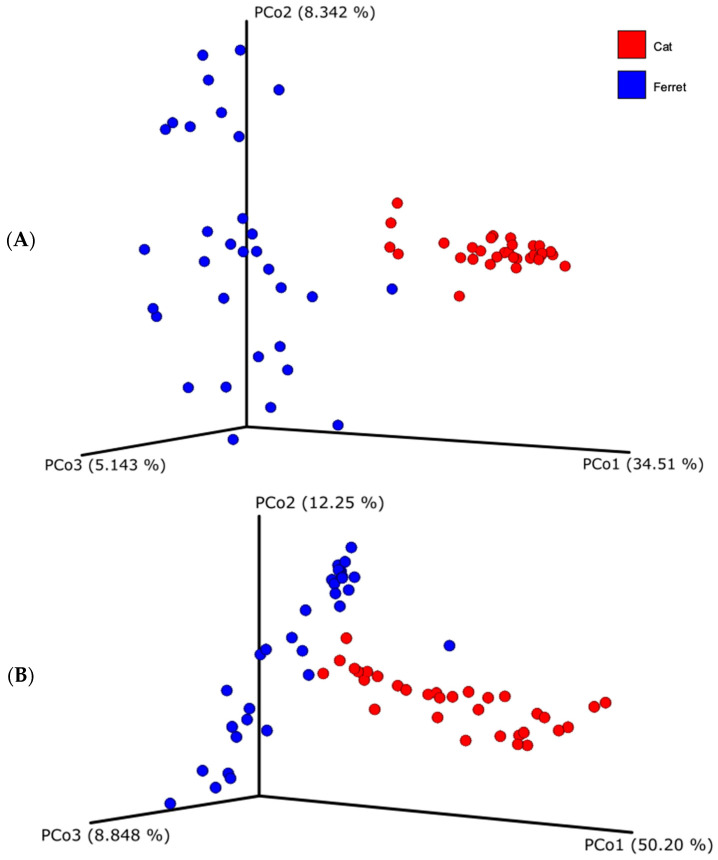
Beta diversity based on unweighted (**A**) and weighted (**B**) Unifrac dissimilarity distance matrices, plotted in a Principal Coordinate Analysis (PCoA). Blue dots represent healthy ferrets and red dots represent healthy cats.

Regarding beta diversity, both unweighted and weighted UniFrac distances showed that the fecal microbial compositions of the ferrets and cats differ substantially (Figure 2). Indeed, the samples from the ferrets, represented with the blue dots, and the samples from the cats, represented with the red dots, are visibly clustered apart. The difference of the beta diversity between the ferrets and cats was tested using a PERMANOVA analysis, which was significant for both UniFrac metrics (weighted UniFrac, *p* = 0.001; unweighted UniFrac, *p* = 0.001).

**Figure 3:**
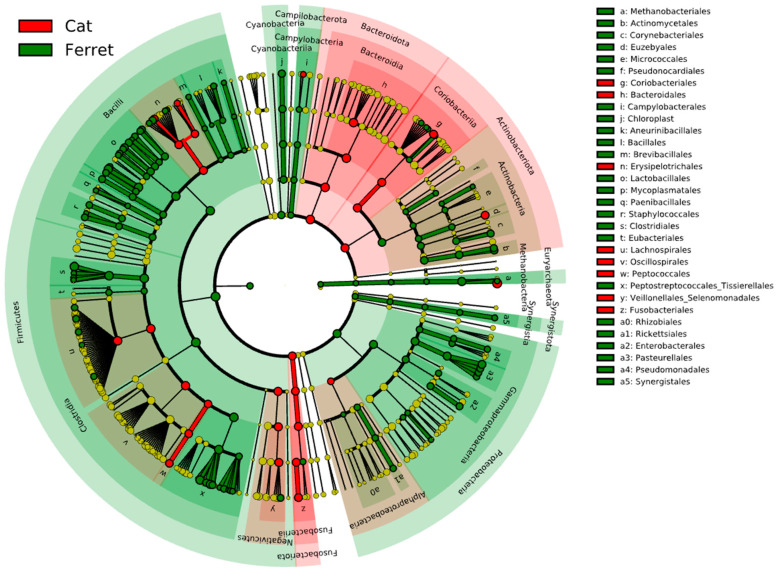
Cladogram reporting the taxonomic representation of statistically and biologically consistent differences (according to LEfSe) between healthy domestic ferrets (highlighted in green) and healthy domestic cats (highlighted in red).

The fecal microbiota of the domestic ferrets and domestic cats are depicted in Figure 3, which reports the results from the LEfSe analysis. The cladogram highlights the taxa that were significantly different based on the animal group. The major difference between the ferrets and cats was found in bacteria in the phylum Firmicutes. The RAs of Staphylococcaceae, Peanibacillales, Mycoplasmatales, Lactobacillales, Clostridiales, and Eubacteriales were significantly more associated with the fecal microbiomes in the ferrets due to a higher relative abundances of these taxa. On the other hand, members of Fusobacteriota were significantly more abundant in the cats compared to the ferrets, as were members of the phylum Bacteroidota and the class Coriobacteriia in the phylum Actinobacteriota.

**Figure 4:**
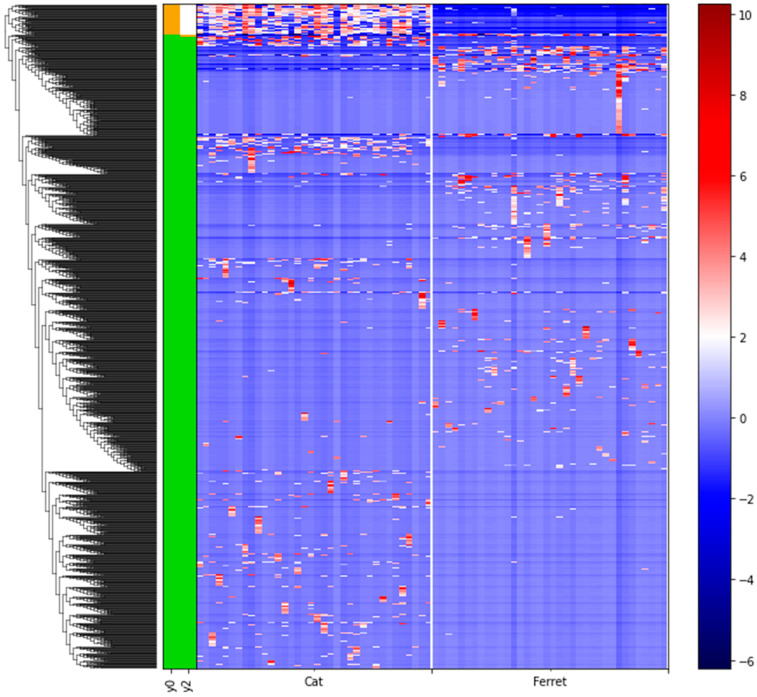
Dendrogram heatmap showing the differential abundances of bacteria between ferrets and cats based on gneiss analysis. A gradient from red to blue is indicating the ASVs log_10_ abundances, from high (red) to low (blue). Green color of the bars is representing the denominator of the balances and the orange color is representing the numerator of the balances.

**Figure 5:**
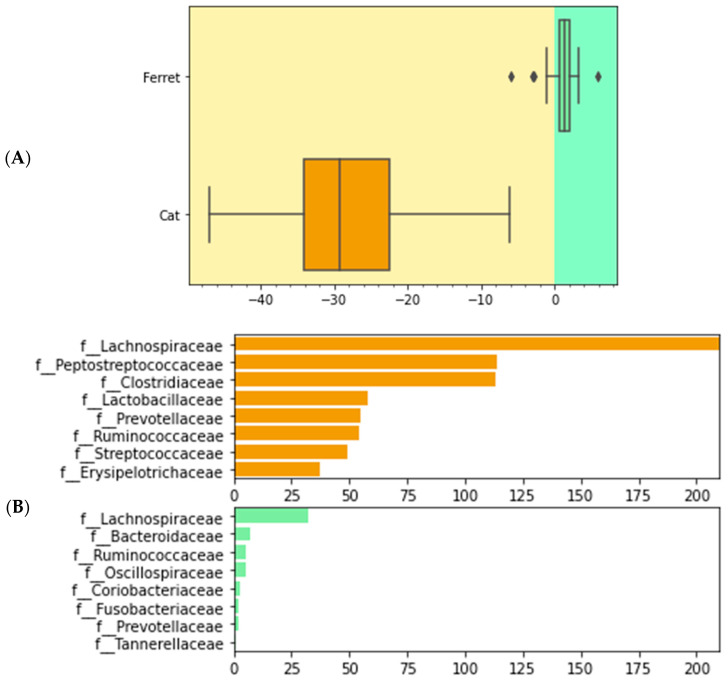
Boxplot of differential abundances of the bacteria parts of the gut microbiomes of healthy ferrets and healthy cats, based on the greatest balances given by the gneiss analysis. Green color represents the denominator of balance y0 and orange color represents the numerator of balance y0. (**A**) Differences of numerator and denominator of balance y0 are grouped by animals. Outliers are represented with the dots. (**B**) Taxa that make up balance y0 are differentiated by numerator and denominator and grouped by species.

We used differential abundance testing using balances in the gneiss analysis to identify bacteria taxa with different RAs within the two groups of animals. In Figure 4, it was represented as a dendrogram heatmap, taking into consideration the y0 and y2 balances. These balances showed the most significant differences between the taxa of the fecal microbiomes of the ferrets and cats. Indeed, it was possible to observe a separation based on the abundances of certain bacteria. Figure 5A is a box plot, grouped by species, to illustrate how well balance y0—shown as the *x* axis—can separate ferrets from cats. The box plot highlights a very clear separation, and the effect size between the ferrets and cats was found to be quite large, indicated by the fact that y0 was the main balance with the largest variance. Figure 5B shows which taxa make up the balance, where the green color represents the denominator of balance y0 and the orange color represents the numerator of the same balance.

## 4. Discussion

Much of the research on domestic ferrets relates to their value as model systems in biomedical research. However, domestic ferrets are also popular companion animals, and veterinarians treating these exotic pets and their owners would like to see advances in how to provide better care for them. The few studies that relate to the microbiomes of domestic ferrets have compared them with other species used in laboratory research. Other companion animals such as dogs and cats are better characterized compared to domestic ferrets. In this study, the fecal microbiota of healthy domestic ferrets (*Mustela putorius furo*) were analyzed and characterized with 16S rRNA sequencing and compared with the fecal microbiomes of a set of healthy cats, with all samples collected, processed, and analyzed using the same methods. Although ferrets are more closely related to other mustelids than to felids, other mustelids are not popular as companion animals. Most diets and medications used in veterinary medicine were developed for more common and traditional companion animals, such as dogs and cats, some of which are repurposed for domestic ferrets.

In this study, we found that the three most abundant phyla in the ferret gut microbiome are Firmicutes, Proteobacteria, and Actinobacteriota, which is an observation that agrees with prior research [10]. Two other articles had the aim of analyzing the intestinal microbiomes of ferrets used in medical research to measure responses to a specific treatment for the reduction in an autistic phenotype in the offspring [8,9]. While the above-mentioned studies were conducted, the authors did not aim to characterize the microbiomes but only aimed to identify differences in the treatment groups or how the bacterial taxa were associated with behavior indices; thus, these findings are difficult to compare with the data described here.

A large and growing body of research has revealed that many health conditions are mediated by the microbiome. As mentioned earlier, insulinoma is a condition that some domestic ferrets develop in middle to late age. Tumors in the pancreas affect insulin production and blood sugar levels and likely impact the gut microbiome as well as digestive function. In addition to the use of prednisolone and/or diazoxide, the medical management of insulinoma includes a dietary intervention. Many clinicians choose feline products, since the efficacy and quality control measures are validated for IBD in cats. The gut microbiome compositions of ferrets have greater similarity to those of minks and sables rather than cats. This observation can be attributed to the shared phylogenetic history of ferrets, minks, and sables within the family Mustelidae, while cats belong to the family Felidae. The mink gut microbiome is characterized by three main phyla, namely Firmicutes, Proteobacteria, and Bacteroidota, with Firmicutes and Proteobacteria constituting more than 90% of the bacterial population [32]. From a study population of minks, it was found that the three most abundant families are Xanthomonadaceae (RAs 15.2%), Lactobacillaceae (RAs 12.8%), and Enterococcaceae (RAs 7.1%). The sable microbiome, on the other hand, is characterized by the presence of Firmicutes, Actinobacteria, and Proteobacteria, which, together, make up more than 95% of the entire microbial population in terms of RAs. The main genera in abundance are instead *Lactobacillus* and *Pseudomonas*, followed by *Arthrobacter*, *Shigella*, and *Leucobacter* [33].

The group of cats used for this study came from a subgroup of cats used for the characterization of the feline microbiome [11]. The results we obtained from this subgroup of feline subjects agree with previous research, both with published studies using the sequencing of amplicons of 16S rRNA regions and those using metagenomic shotgun sequencing. A recent study reported the characterization, through the analysis of the V4 16S rRNA gene region, of the gut microbiome from a healthy dataset of cats, providing ranges for the bacterial community structure in a healthy population. In this study, it was observed that the most abundant genera in descending order were *Prevotella*, *Bacteroides*, *Collinsella*, *Blautia*. and *Megasphaera*, while the most prevalent ones in descending order were *Bacteroides*, *Blautia*, *Lachnoclostridium*, *Sutterella*, and *Ruminococcus gnavus* [11]. Studies that apply whole-genome shotgun sequencing techniques also confirm what has been currently observed. According to Ma et al. (2022) [34], the cat gut microbial population at the phylum level is composed of five main phyla, with Firmicutes (47.7%) first, followed by Bacteroidetes (27.1%), and subsequently followed by Actinobacteria, Proteobacteria, and Fusobacteria. Among the most abundant genera, the top five were *Prevotella*, *Clostridium*, *Collinsella*, *Blautia*, and *Bacteroides*, in order of abundance, and in general, the top 20 genera also included *Ruminococcus*, *Megasphaera*, and *Lachnoclostridium*. These results, in addition to being in agreement with the ones described previously in the literature, are also in agreement with other studies of 16S rRNA gene analysis, such as that of Fischer et al. (2017) [35].

Given the digestive anatomy, it would be expected that ferrets would have similar nutrient requirements to other carnivores, such as cats, but due to their shorter colon, they might have less ability to digest and absorb some nutrients. Fox et al. [16] found that domestic ferrets had lower crude protein contents and higher fat digestibility compared with cats that were fed the same diet. Due to these observations, a unique bacterial population would be expected to occur in domestic ferrets compared to domestic cats.

## 5. Conclusions

In conclusion, although the domestic ferret and the domestic cat are both obligate carnivores, this study shows how, from a microbiome perspective, these two species are very different. First, the fecal microbiome of the ferret is significantly less complex in terms of bacterial diversity compared to that of the domestic cat. Second, bacterial composition of fecal microbiomes collected from the two species differed substantially. As a result, it is likely that in the case of gastroenterological pathology or intestinal dysbiosis, the ferret may require different care to modulate the microbiome. These results are not surprising given the short transit time in ferrets compared to cats, providing less opportunity time for microbiome fermentation.

## Figures and Tables

**Table 1:** Comparison of the Relative Abundances (RAs) of the most common phyla found in healthy ferrets and healthy cats. Gradient of color (dark to light) highlights the three main Phyla, in terms of RAs, of ferrets and cats.

	Ferrets (%)		Cats (%)	
	Mean	Median	Mean	Median
Firmicutes	90.4	97.2	64.6	64.3
Proteobacteria	4.8	0.6	2.4	1.7
Actinobacteriota	3.2	0.8	10.2	8.7
Bacteroidota	0.7	0.02	19.2	17.6
Fusobacteriota	0.5	0.06	3.1	1.1
Cyanobacteria	0.2	0.01	0.03	0.0
Desulfobacterota	0.005	0.00	0.35	0.04

**Table 2:** The most prevalent core taxa identified in the gut microbiome of domestic pet ferrets (found in ≥50% of the healthy reference population).

Phylum	Class	Order	Family	Genus	Mean	Median	SD	Prev. (%)
Firmicutes	Bacilli	Lactobacillales	*Streptococcaceae*	*Streptococcus*	15.106	4.317	20.55	100.00
Firmicutes	Clostridia	Clostridiales	*Clostridiaceae*	*Clostridium sensu stricto 1*	28.789	19.748	30.15	100.00
Firmicutes	Bacilli	Lactobacillales	*Lactobacillaceae*	*Lactobacillus*	4.179	0.365	10.54	94.44
Firmicutes	Clostridia	Peptostreptococcales-Tissierellales	*Peptostreptococcaceae*	*Romboutsia*	13.131	3.570	21.95	94.44
Actinobacteriota	Actinobacteria	Corynebacteriales	*Corynebacteriaceae*	*Corynebacterium*	0.645	0.080	1.67	86.11
Firmicutes	Bacilli	Lactobacillales	*Enterococcaceae*	*Enterococcus*	3.045	0.222	8.18	86.11
Firmicutes	Bacilli	Lactobacillales	*Streptococcaceae*	*Lactococcus*	2.122	0.171	4.48	86.11
Firmicutes	Clostridia	Peptostreptococcales-Tissierellales	*Peptostreptococcaceae*	*Peptostreptococcus*	0.593	0.198	0.98	83.33
Actinobacteriota	Actinobacteria	Actinomycetales	*Actinomycetaceae*	*Actinomyces*	1.671	0.091	3.19	80.56
Firmicutes	Clostridia	Peptostreptococcales-Tissierellales	*Peptostreptococcaceae*	*Paeniclostridium*	9.263	3.178	14.90	80.56
Proteobacteria	Gammaproteobacteria	Enterobacterales	*Enterobacteriaceae*	*Escherichia-Shigella*	1.173	0.045	3.10	77.78
Fusobacteriota	Fusobacteriia	Fusobacteriales	*Fusobacteriaceae*	*Fusobacterium*	0.500	0.058	1.77	75.00
Cyanobacteria	Cyanobacteriia	Chloroplast	*Chloroplast*	*Chloroplast*	0.223	0.015	0.76	69.44
Firmicutes	Bacilli	Lactobacillales	*Leuconostocaceae*	*Leuconostoc*	0.239	0.014	0.53	63.89
Firmicutes	Clostridia	Lachnospirales	*Lachnospiraceae*		0.271	0.027	0.62	61.11
Firmicutes	Bacilli	Lactobacillales	*Leuconostocaceae*	*Weissella*	0.068	0.009	0.19	58.33
Firmicutes	Clostridia	Peptococcales	*Peptococcaceae*	*Peptococcus*	0.210	0.013	0.65	58.33
Firmicutes	Bacilli	Lactobacillales	*Aerococcaceae*	*uncultured*	0.112	0.017	0.24	52.78
Firmicutes	Bacilli	Lactobacillales	*Vagococcaceae*	*Vagococcus*	0.299	0.016	0.55	52.78
Firmicutes	Bacilli	Staphylococcales	*Gemellaceae*	*Gemella*	0.309	0.003	1.28	52.78
Firmicutes	Clostridia	Peptostreptococcales-Tissierellales	*Peptostreptococcales-Tissierellales*	*Gallicola*	0.094	0.001	0.16	50.00
Firmicutes	Negativicutes	Veillonellales-Selenomonadales	*Veillonellaceae*	*Veillonella*	0.237	0.001	0.86	50.00

## Data Availability

The raw data of the ferrets have been uploaded and are available on the NCBI Sequence Read Archive (Bioproject ID PRJNA988157). The raw data of the cats are not available publicly due to their commercial value, but they are available upon request for academic use only.

## Supplementary Materials

The following supporting information can be downloaded at https://www.mdpi.com/article/10.3390/ani13213354/s1, Table S1. Characteristics of ferrets included in the healthy population set (n = 36).
