# Supplementary material for: Characterization and Description of the Fecal Microbiomes of Pet Domestic Ferrets (Mustela putorius furo) Living in Homes
Source: Animals (Basel). 2023 Oct 29;13(21):3354. doi: 10.3390/ani13213354 (PMC10647649; doi:10.3390/ani13213354)
Supplement: Supplementary file 1 [file animals-13-03354-s001.zip › animals-2627287-supplementary.pdf]

Supplementary Table S1. Characteristics of ferrets included in the healthy population set (*n* = 36).

N. ferrets	Mean Age (years)	Range Age (years)	Body wieght (kg)	Female	Male	Spayed or Neutered	Kibble	Dehydrated	Raw
36	3.13	1-9	2.35	13	22	16	35	28	28
